# Point of Care Ultrasound Accurately Distinguishes Inflammatory from Noninflammatory Disease in Patients Presenting with Abdominal Pain and Diarrhea

**DOI:** 10.1155/2016/4023065

**Published:** 2016-04-20

**Authors:** Kerri L. Novak, Deepti Jacob, Gilaad G. Kaplan, Emma Boyce, Subrata Ghosh, Irene Ma, Cathy Lu, Stephanie Wilson, Remo Panaccione

**Affiliations:** ^1^Inflammatory Bowel Disease Clinic, Division of Gastroenterology and Hepatology, University of Calgary, Calgary, AB, Canada T2N 2T9; ^2^Department of Community Health Sciences, Calgary, AB, Canada T2N 2T9; ^3^Division of General Internal Medicine, Calgary, AB, Canada T2N 2T9; ^4^Division of Gastroenterology, University of Alberta, Edmonton, AB, Canada T2N 2T9; ^5^Diagnostic Imaging, University of Calgary, Calgary, AB, Canada T2N 2T9

## Abstract

*Background*. Approaches to distinguish inflammatory bowel disease (IBD) from noninflammatory disease that are noninvasive, accurate, and readily available are desirable. Such approaches may decrease time to diagnosis and better utilize limited endoscopic resources. The aim of this study was to evaluate the diagnostic accuracy for gastroenterologist performed point of care ultrasound (POCUS) in the detection of luminal inflammation relative to gold standard ileocolonoscopy.* Methods*. A prospective, single-center study was conducted on convenience sample of patients presenting with symptoms of diarrhea and/or abdominal pain. Patients were offered POCUS prior to having ileocolonoscopy. Sensitivity, specificity, positive predictive value (PPV), and negative predictive value (NPV) with 95% confidence intervals (CI), as well as likelihood ratios, were calculated.* Results*. Fifty-eight patients were included in this study. The overall sensitivity, specificity, PPV, and NPV were 80%, 97.8%, 88.9%, and 95.7%, respectively, with positive and negative likelihood ratios (LR) of 36.8 and 0.20.* Conclusion*. POCUS can accurately be performed at the bedside to detect transmural inflammation of the intestine. This noninvasive approach may serve to expedite diagnosis, improve allocation of endoscopic resources, and facilitate initiation of appropriate medical therapy.

## 1. Introduction

Irritable bowel syndrome (IBS) is a functional gastrointestinal (GI) disorder, characterized by chronic abdominal pain and altered bowel habit, with a benign natural history. With a worldwide prevalence approximating 10–15%, it is a common disorder and one of the most frequent sources of referral to gastroenterology [1]. The potential economic burden of IBS is significant, and some patients have long-term persistence of symptoms with need for more chronic management strategies [1–3]. In contrast, the incidence of the inflammatory bowel disease (IBD) in North America is only a fraction of IBS [4].

A subset of patients with IBS have diarrhea and abdominal pain as predominant symptoms, presenting similarly to those with inflammatory bowel disease (IBD), thus making the clinical distinction challenging based on symptoms alone [5, 6]. Timely differentiation of IBD from IBS is critical because delay in diagnosis of IBD can increase the risk of developing complications, hospitalization for management, and surgery [7].

Clinical indices used to accurately identify inflammation in patients with symptoms exist but are not discerning for IBD and have not been incorporated into routine diagnostic investigation [8]. Laboratory markers are also not always consistent predictors of inflammation to exclude IBD [9]. For example, fecal inflammatory markers may exclude inflammation present in the colon but are not always reliable for small bowel disease common in Crohn's disease [10]. Endoscopy represents the gold standard diagnostic modality to investigate symptoms that suggest inflammation. Endoscopic resources are limited, however, and the number of patients with abdominal symptoms diagnosed at endoscopy with IBD is low [5]. In addition, current guidelines suggest that many patients should be managed conservatively with symptom-based diagnosis, given the low yield of traditional means to detect organic disease [11, 12]. Alternate, noninvasive diagnostic strategies such as computed tomography (CT) and magnetic resonance imaging (MRI) are not routinely recommended for the investigation of suspected IBS, yet these strategies are increasingly used [13]. Screening with these modalities for inflammation or other worrisome etiology in IBS should be limited, given the concern over radiation exposure with CT and the high cost and limited access to MRI [13]. Ultrasound (US) is a noninvasive, accurate, safe, effective, and easily accessible modality. It can be performed in clinic by gastroenterologists as an extension of the physical examination [14].

The use of point of care ultrasound (POCUS) for noncardiac purposes in medicine by nonradiologists has increased significantly in the last decade [15]. Focused examination performed at the bedside during routine clinical assessment may be used to answer binary diagnostic questions: “does this patient have free fluid in the abdomen?” [16]. Although widely implemented as an integral part of routine evaluation for patients with established IBD in many parts of Europe, it is not widely used as a diagnostic tool by gastroenterologists in North America. The aim of this prospective study of patients investigated for symptoms to exclude organic disease was to evaluate the diagnostic accuracy of gastroenterologist performed POCUS in excluding luminal inflammation relative to gold standard ileocolonoscopy. This tool may be used in the future to help guide the appropriate use of diagnostic endoscopy.

## 2. Materials and Methods

### 2.1. Patient Population and Study Design

Consecutive patients presenting for assessment of symptoms including abdominal pain and diarrhea suspicious for IBD to the University of Calgary GI clinic were selected for this pilot study from January 1, 2013, to June 1, 2015. Patients with significant abdominal obesity (BMI > 40), pregnancy, liver disease with ascites, or conditions of previously known luminal inflammation were excluded. Informed consent was obtained prior to the examination and the study was approved by the Conjoint Health Research Ethics Board guidelines.

### 2.2. Clinical Assessment and POCUS Examination

All patients underwent a physical examination as per standard of care. Laboratory inflammatory markers including C-reactive protein (CRP) were recorded where available, within 2 months of POCUS. Fecal calprotectin was not available at the time of the study at this center. A gastroenterologist (KN), with formal training in US of the GI tract and 3-year experience in the modality, conducted a focused luminal POCUS examination. A standardized approach was used, conducting the examination from the left lower quadrant, examining the colon from the rectum to the cecum, with examination of the rectum, sigmoid, descending, transverse, and ascending colon, and cecum. The terminal ileum was then evaluated, followed by systematic four-quadrant examination to include the remaining small bowel. All exams were completed using a Phillips IU22 US machine utilizing a range of transducers, including high frequency curved (4–9 mHz) and linear probes (12–15 mHz).

Presence of inflammatory activity was documented in binary fashion (“active”/“inactive”). Inflammation was deemed to be present, as per standard sonographic assessment, if there was increased bowel wall thickness (>3 mm for small bowel, >4 mm for large bowel) and the presence of any additional established indicators of inflammation: mesenteric inflammatory fat, lymph nodes, and hyperemia or blood flow as detected on color Doppler imaging [17]. The anatomic site(s) of disease was also recorded. In addition, complications including luminal stenoses, penetrating complications or fistulas, perianal disease, and intra-abdominal abscesses were described and recorded if present. Finally, commentary was made regarding echo-stratification or preservation of wall layers as well as the overall quality of the examination (adequate/inadequate).

Patients with normal blood work, negative endoscopy, and histology and who met Rome III criteria for IBS were identified as having IBS (diarrhea predominant) [18]. Those with normal endoscopic features were diagnosed with microscopic colitis, if confirmation was made histologically [19]. Inflammatory bowel disease (IBD) was diagnosed/confirmed where both endoscopic and histologic markers were present; in these cases anatomic locations and patterns were recorded. The gold standard comparator was endoscopy alone for all studies. Finally, alternate etiologies including segmental colitis associated with diverticula, infectious, or ischemic colitis were also recorded.

### 2.3. Endoscopic and Histologic Examination

All patients underwent ileocolonoscopy after POCUS. The time interval to routine or expedited endoscopy, determined based on clinical suspicion of disease, was recorded. Extent of the endoscopy was recorded including intubation of the terminal ileum or lack of as well as the presence of disease activity, which was recorded in a binary fashion (present/absent), along with documentation of the etiology of the inflammation (confirmation of Crohn's disease or ulcerative colitis or other suspected etiology) and, if present, the anatomic location. Where clinically indicated, biopsies were taken in both the small and large bowel and results (presence/absence of inflammation) were recorded. If biopsies were taken as part of the standard evaluation, this too was recorded as confirmation of organic disease or its absence.

### 2.4. Patient Satisfaction

A subset of patients (14/58) completed a 12-question patient satisfaction questionnaire, with graded responses following the POCUS examination in clinic. The questionnaire evaluated patient satisfaction with the overall experience, patient perception of the value of POCUS, and the willingness to incorporate POCUS as an extension of the physical examination in clinic.

### 2.5. Statistical Analysis

Demographic data and ultrasound quality were evaluated using descriptive statistics. Further analysis was aimed at evaluating the ability of POCUS to detect inflammation in symptomatic patients compared with standard colonoscopy; again, colonoscopy was considered the gold standard. Sensitivity, specificity, positive predictive value (PPV), negative predictive value (NPV), and the positive and negative likelihood ratios (LR) with 95% confidence intervals (CI) were calculated for the overall POCUS score, compared to endoscopy only. To further evaluate POCUS, the above-mentioned statistics were stratified for segmental analysis, either ileum or colon. Accuracy of the detection of disease location, if present, was also evaluated. In cases where the ileum was not seen in endoscopic examinations, correlations were made between the endoscopic and US segments of the colon alone. Finally, the ultrasound examination quality was measured descriptively. Histology was reported; however, it was not used as the gold standard.

## 3. Results

A total of 58 patients presenting to the clinic with symptoms were included. Demographic details are presented in Table 1. Two patients with a high BMI (>35) contributed to poor exam quality; however, the bowel was visualized; thus, they were included in the analysis. The remaining 56/58 (96%) examinations were of good quality. Endoscopies were performed following POCUS with a median of 30 days and all were included in the analysis. All but 2 examinations intubated the terminal ileum (Table 1).

**Figure 1:**
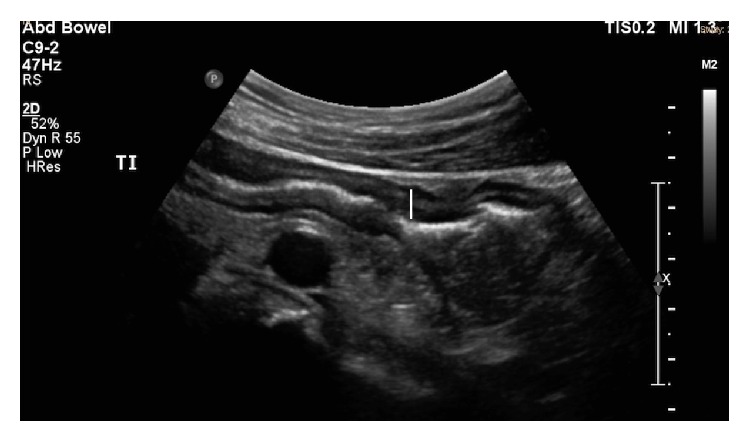
First suggestion of terminal ileal Crohn's disease identified on sonography, with thickened distal ileum running over the hypoechoic iliac artery. The white line marks the thickened ileal wall with echogenic or white air in the lumen.

**Figure 2:**
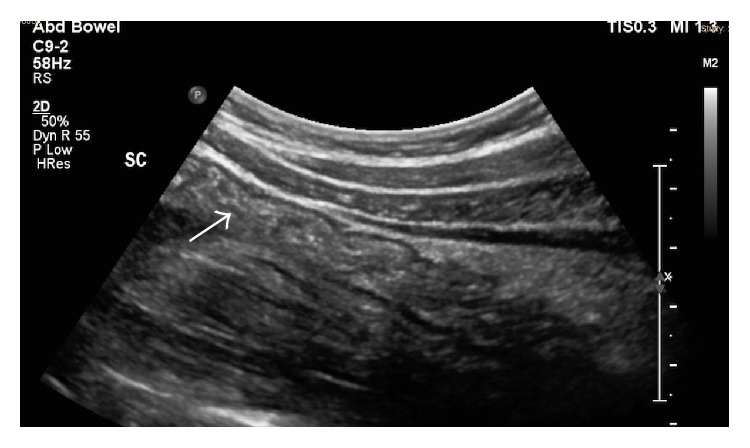
Normal sigmoid colon with normal haustral folds (white arrow).

Inflammatory activity was detected sonographically (positive study) in 9/58 (15.5%) ranging from mild to severe inflammation (Tables 2 and 3). Mesenteric lymph nodes and increased bowel wall thickness were the most common US parameters identified and were seen in all patients with US activity (9/9 100%) (Figure 1). The only complication identified was a stricture, present in 1/9 cases (11%), characterized by fixed luminal apposition and narrowing, with evidence of proximal small bowel dilation. Ten (10/58 or 17%) patients had inflammation confirmed on endoscopy (Tables 3 and 4). Most patients (93%) had biopsies and pathology completed, and all 10 had inflammation confirmed histologically. Of these, 4/10 (40%) had ileal CD (Figure 1), 1 with ileocolonic distribution of inflammation, 5 had colonic inflammation (1 with a new diagnosis of ulcerative colitis, 1 with segmental diverticular inflammation, and the remaining 3 had patchy, mild colonic inflammation consistent with colonic CD). There was one false-positive US exam, with 2 false-negative findings (see 2 × 2 data in Table 3). In addition, 9 patients with normal colons both sonographically and endoscopically revealed microscopic colitis on pathology. One patient exhibited no evidence of disease endoscopically nor on US but had mild inflammatory activity identified histologically in the ileum, suggesting possibly early IBD. All other cases confirmed diarrhea-predominant IBS (38/58 65.5%) (Figure 2). There were no cases of ulcerative colitis identified in this study. In those patients with a positive POCUS, endoscopic exams occurred with shorter intervals with a median of days. The shortest wait time for endoscopy after US was endoscopy on the same day of the US exam.

POCUS exhibited a PPV of 88.9% (95% CI: 51.75–99.72) and a NPV of 95.7% (95% CI: 85.5–99.5). The overall sensitivity and specificity of POCUS were 80% (95% CI: 44.4–97.5) and 97.8% (95% CI: 88.5–99.9), respectively, using a cutoff for bowel wall thickness of 3 mm for the ileum and 4 mm for the colon, with positive and negative LRs of 36.8 and 0.20 (Table 3). When the results were analyzed by segment, comparing colonic disease and ileal disease separately, ultrasound detection of ileal disease exhibited higher sensitivity compared to the colon compared to gold standard endoscopy (Table 3).

Of the 10 patients with confirmed inflammatory activity on endoscopy, all had CRP (7/10 within 1 month) completed, and 5 were elevated while the other 5 were within normal range. The median CRP value for these 10 patients was 6.9 mg/L. In those with elevated CRP, none of the US examination, confirmed endoscopically, revealed severe disease while all those 3 cases with moderate to severe sonographic disease activity had CRP within normal range, measured within 40 d of the US.

All 14 patients who completed questionnaires described the preparation for US as “easy” and reported an increased understanding of their disease and disease location following the exam. All of these patients believed POCUS had value in guiding disease management and found the increased clinic time acceptable and beneficial to their health.

## 4. Discussion

To our knowledge, this is the first prospective study to evaluate the accuracy of gastroenterologist-performed POCUS in excluding inflammation in symptomatic patients compared to colonoscopy in North America. The accuracy established here is similar to previous published data [20–22]. There is a significant need for noninvasive, safe, resource-conscious modalities of accurately detecting inflammation in symptomatic patients, as symptoms alone do not accurately reflect inflammatory disease activity and incorrect diagnosis can delay appropriate management of IBD [23, 24]. Ultrasound has been shown to significantly impact clinical decisions, when used at the bedside [25]. Symptoms of diarrhea and abdominal pain are common as manifestations of irritable bowel syndrome, and diarrhea is a symptom that often drives patient visits to primary care [1, 21]. Commonly used noninvasive markers such as C-reactive protein (CRP) may be falsely negative and fecal calprotectin has variable availability and may have a lower accuracy in detecting small bowel inflammation [9, 26].

The rate of pathologically proven histologic inflammation in this study, either microscopic colitis or inflammatory bowel disease, was 20/58 (34%). This figure is much higher than that quoted for larger populations of patients with IBS including all subtypes of IBD. Given their symptoms, this population was identified as “high risk” and in need of ileocolonoscopy as part of standard of care investigation and the high pathology rate likely reflects some bias or overrepresentation of pathology, given the convenience sampling from the “high-risk IBD” clinic. However, gold standard evaluation with endoscopy is not always indicated in IBS patients, as it is resource intensive and carries the associated risks of an invasive examination (including patient tolerance) [12, 22]. Tools to support indication for endoscopy would be helpful, in addition to the timing/urgency of endoscopy. In this study, 83% (48/58) of patients had no evidence of inflammation on POCUS or endoscopy, suggesting that ultrasound may obviate the need for endoscopy in select patients, especially in combination with a low CRP, as recent evidence suggests that CRP < 1.0 makes the probability of IBD very low [9]. Clinical factors suggesting organic etiology including MC may also be useful, such as night time stooling, weight loss, and age greater than 50 years [27]. In this study, CRP was not suggestive of more severe disease either sonographically or endoscopically, illustrating, even in this small sample, limitations with using CRP as a guide for the need for endoscopy. Given current wait-times for nonurgent endoscopy, inappropriate delays may result as a consequence of false-negative results of currently available tests [24]. Thus, a widely available, accurate set of clinical tools that would include noninvasive US may guide the appropriate use of diagnostic endoscopy. This has been demonstrated here with the high likelihood ratio of US, suggesting a very high probability of confirming IBD with a positive scan [28]. This may allow for confident expedition of urgent diagnostic assessment and early appropriate therapy. Here, abnormal US findings resulted in expedited endoscopy with median time to diagnosis of 26 days in those with active IBD on sonography, compared to a median time to endoscopy of 30 days for the study population.

The accuracy of detecting disease in the terminal ileum on cross-sectional imaging tends to be greater compared to the colon, as was exhibited in this study on US [14]. The sensitivity and specificity for detecting ileal disease was higher compared to colonic disease (Table 4). Both cases (2/10) missed on US were mild endoscopically, possibly not yet exhibiting cross-sectional disease activity [29]. Regardless of imaging modality, mild disease is difficult to detect, given the absence of transmural involvement, as early disease involves the mucosa and this is also reflected here, as there were 2 false negatives versus only 1 false positive (Table 3). This circumstance may be best served by the addition of fecal calprotectin, valuable for detecting inflammation in this context [9]. Although a normal US may not obviate the need for endoscopic confirmation, it was however reassuring and suggested a need for a less urgent endoscopy as any disease missed was most likely relatively mild. There were no cases where the distal terminal ileum was normal on endoscopy and disease was isolated to the proximal small bowel, as has been reported in up to 30% of cases with small bowel involvement [30]; however, in 2 patients with both US and endoscopic activity (true positives), there was an estimation between 25 and 30 cm of active disease proximal to that region examined endoscopically. Thus, US also provided insight into the length or burden of the disease [31]. One case exhibited negative US and negative endoscopy, yet mild inflammatory activity on pathology. This may indicate early inflammatory bowel disease, an important etiology to detect early in order to alter the natural disease history. This again highlights the need for concomitant clinical tools such as stool and serum-based inflammatory markers, combined with clinical factors, to direct appropriateness of endoscopic evaluation and biopsies.

Nine patients with both negative US and endoscopy had microscopic colitis (MC) confirmed histologically. Five of these patients (55%) exhibited extensive small bowel fluid filled loops, a novel finding suggesting possible pathology of the small bowel as well (Figure 2). This requires investigation and confirmation with larger numbers. Small bowel involvement in MC is not well understood and may be missed on endoscopy because the small bowel may not be extensively visualized or biopsied. Thus, POCUS may also contribute significant understanding of anatomy and function in regions of the bowel not seen endoscopically.

Image quality has been suggested to be more variable in US compared to MR or CT, potentially limiting US in some populations, specifically in those with high BMI (>35) with increased abdominal wall adiposity. Only 2 patients in this study had poor quality examinations due to high BMI; however, the US examinations were still deemed sufficient. There are no currently existing training guidelines for gastroenterologists to guide minimal requirement for competency in luminal sonography in Canada, given the evolution of this emerging modality in the field. However, it is a standard part of training in many European countries including Germany and Italy, where at least 100 examinations are recommended [32]. Inexperienced sonographers have also shown to have significant ability to accurately detect disease [33].

Patient engagement and satisfaction are important components of establishing rapport and trust and are crucial for long-term success in the management of both chronic functional disorders and inflammatory conditions. US improved patient understanding and was well tolerated and was generally preferred over invasive endoscopy. This tool may prove to be important in facilitating understanding and reassurance for patients at diagnosis and is a radiation-free and relatively inexpensive modality that can be used serially for disease monitoring in established IBD. The sample reflecting patient satisfaction was however small, in this early pilot study.

There are a number of limitations in this study, as it is small and is limited to one academic center with one sonographer performing bedside US examinations. These limitations should of course be kept in mind when considering the generalizability of this study to other centers. This is, however, a novel modality in gastroenterology, and training guidelines along with reporting and quality frameworks must be established. In addition, this preliminary analysis is underpowered; therefore, a single error, false-positive or negative, will significantly adversely impact the sensitivity and specificity in this small sample. Thus, prospectively collected larger studies are necessary to understand accuracy of the modality with and without additional clinical factors such as clinical predictors and inflammatory markers.

The time interval between US and endoscopy was variable with few endoscopic examinations completed more than 3 months after the POCUS. This likely reflects real life practice, however, as these patients were clinically suspected to have IBS and investigation for most of them was triaged as nonurgent. There were no medical interventions initiated for any patients; therefore, no structural changes should result between imaging and endoscopic evaluations. Finally, the sonographer in this study was also performing ileocolonoscopy, not blinded to the US results, which may bias the commentary on endoscopy. In order to minimize this bias, nearly all (94%) had biopsies taken to definitively exclude unrecognized inflammation.

## 5. Conclusion

Point of care US is a safe, noninvasive means of accurately detecting inflammation in patients being investigated for symptoms of diarrhea and abdominal pain. It is timely, as it occurs at the bedside during clinical assessment. It is well accepted by patients and can be employed to further direct definitive diagnosis and management and possibly obviate the need for endoscopic evaluation in certain cases. Given the high positive likelihood ratio with a positive exam, it can be effectively used as a triage tool to expedite endoscopy.

## Figures and Tables

**Table 1:** Patient demographic and laboratory investigation data (change title).

	*n* (%)
Gender	
Male	19 (33)
Female	39 (67)
Median age (years)	32.8 (17.8–72.4)
Median CRP (mmL, range) (*n* = 31)^*∗*^	3.4 (0.6–36.7)
Median time between POCUS and CRP (days)	39.7 (0–127)
Median time from POCUS to endoscopy (days, range)	30 (0–149)
Adequate POCUS exam quality	56 (96.5)
TI intubation on endoscopy	56 (96.6)

^*∗*^Only a subset had CRP measured that is the *n* = 31.

**Table 2:** Descriptive ultrasonographic and endoscopic data.

	Ultrasound *N* (%)
Active inflammation on US	9/58 (15.5)
Increased bowel wall thickness	9/9 (100)
Lymph nodes	9 (100)
Inflammatory fat	7 (78)
Hyperemia	3 (33)
Preserved wall layers	10 (100)
Complications^+^	1 (11%)
Active site on endoscopy (*n* = 10)	
Ileum	4 (40%)
Colon	5 (50)
Ileocolonic	1 (10%)

^+^Complications identified include any of abscess, stricture, phlegmon, or inflammatory mass.

**Table 3:** A 2 × 2 table for ultrasound compared to gold standard endoscopy.

	Positive endoscopy	Negative endoscopy
Positive US	8	2
Negative US	1	47

**Table 4:** Sensitivity, specificity, PPV, and NPV of POCUS relative to endoscopy.

	Overall	Ileum	Colon
Sensitivity% (CI%)	80.0 (44.4–97.5)	100.0 (47.8–100.0)	60.0 (914.7–94.7)
Specificity% (CI%)	97.8 (88.5–99.9)	98.2 (90.45–99.9)	100.0 (93.3–100.0)
PPV% (CI%)	88.9 (51.7–99.7)	83.3 (35.9–99.6)	100.0 (29.2–100.0)
NPV% (CI%)	95.7 (85.5–99.5)	100.0 (93.5–100.0)	96.36 (87.5–99.6)
Positive LR (CI%)	36.8 (5.2–262.1)	56.0 (8.0–390.6)	— (—)
Negative LR (CI%)	0.20 (0.06–0.71)	— (—)	0.40 (0.14–1.17)
